# Stress-induced nuclear GAPDH: Scientific update and clinical application

**DOI:** 10.1016/j.neurot.2025.e00725

**Published:** 2025-09-03

**Authors:** Parimala Vedula, Koko Ishizuka, Arisa Hayashida, Kota Sueo, Akira Sawa

**Affiliations:** a Departments of Biomedical Engineering, Johns Hopkins University School of Medicine, Baltimore, MD 21205, USA; b Departments of Psychiatry, Johns Hopkins University School of Medicine, Baltimore, MD 21205, USA; c Departments of Neuroscience, Johns Hopkins University School of Medicine, Baltimore, MD 21205, USA; d Departments of Pharmacology, Johns Hopkins University School of Medicine, Baltimore, MD 21205, USA; e Departments of Genetic Medicine, Johns Hopkins University School of Medicine, Baltimore, MD 21205, USA; f Department of Mental Health, Johns Hopkins University Bloomberg School of Public Health, Baltimore, MD 21205, USA

**Keywords:** Glyceraldehyde 3-phosphate dehydrogenase (GAPDH), Moonlighting protein, Nucleus, Stress response, Cellular homeostasis, Posttranslational modification, *S*-nitrosylation, Therapeutics

## Abstract

Glyceraldehyde 3-phosphate dehydrogenase (GAPDH) is known as a moonlighting protein beyond its original glycolytic function. Stress-induced nuclear translocation of GAPDH has been reproducibly reported, which results in variety types of cellular responses, including cell death and dysfunction. Blocking this stress-induced cascade has been regarded as a target of drug discovery and development for human disease conditions, particularly for neurological and psychiatric diseases. There are promising small compounds that can block this cascade in cell and animal models. Nevertheless, the clinical trials for Parkinson's disease and amyotrophic lateral sclerosis with one of these compounds Omigapil were unsuccessful. Including these failure cases, this review article discussed the scientific frontline of GAPDH, particularly stress-induced nuclear GAPDH, and its potential for clinical applications.

## Introduction: moonlighting protein GAPDH

Glyceraldehyde 3-phosphate dehydrogenase (GAPDH) is originally known as one of the enzymes in the glycolytic pathway, which is central for cellular homeostasis [1,2]. More recent studies on GAPDH have deciphered its non-glycolytic functions [[1], [2], [3], [4], [5], [6]]. Nowadays, GAPDH is recognized as one of the most representative “moonlighting” proteins [7].

One of the most well-studied non-glycolytic features associated with GAPDH is its nuclear translocation under stressed conditions [[1], [2], [3]]. Multiple groups have studied the role of stress-induced nuclear GAPDH in neuronal and non-neuronal contexts, which is spanning from basic molecular and cellular studies to clinical trials [1,2,8,9]. Another non-glycolytic feature of GAPDH is its binding with ribonucleic acids (RNAs) and deoxyribonucleic acids (DNAs) [10,11]. For example, the interaction of GAPDH with AU-rich elements within the 3′ UTR of interferon (IFN)-γ mRNA contributes to the posttranscriptional control of T cell effector function [5].

The goal of this review article is to discuss the translational potential of GAPDH for neurological and psychiatric disorders. The blockade of stress-induced nuclear GAPDH and resultant neuronal death by medicines that interfere with nuclear GAPDH has been expected to be a promising route for translation. In the first half of this article, we will summarize basic research on stress-induced nuclear GAPDH, followed by the discussion on what is known and what is unknown about nuclear GAPDH. In the second half, we will review past clinical trials that used medicines against stress-induced nuclear GAPDH and discuss the future perspectives.

## Nuclear translocation of GAPDH in response to stressors

### Nuclear GAPDH: a classic view

Classically the existence of glycolytic proteins has been reported not only in the cytoplasm but also in the nucleus [1,2]. In the past several decades, the interaction of GAPDH with DNAs has been reported by multiple groups [11]. Particularly, GAPDH binds to both single- and double-stranded telomeric DNA and has been recently shown to protect against rapid telomere shortening induced by chemotherapeutic drugs that release ceramides [12]. In contrast, another study demonstrated that the lysine residue in the catalytic site of GAPDH binds to the telomerase RNA component (TERC), leading to the inhibition of telomerase activity, telomere shortening, and the promotion of cellular senescence [13]. Nevertheless, many outstanding questions remain elusive: for example, it is unclear whether nuclear GAPDH exists constitutively present or context-dependently. It also remains elusive how GAPDH is sorted to the nucleus.

### Nuclear GAPDH for cell death in neurons and non-neuronal cells

Following an initial finding that antisense oligonucleotide treatment against GAPDH can block cell death of primary neuron cultures [14], another group reported that nuclear translocation of GAPDH participates in stress-induced cell death in both non-neuronal and neuronal cells [15]. These stressors include oxidative stress, inflammatory signaling activators, serum starvation, and genotoxin [6,15]. Hara et al. [6] demonstrated that these stressors elicited *S*-nitrosylation (or possibly other nitrosative or oxidative posttranslational modifications) of GAPDH on cysteine-150 in rat/cysteine-152 in human (Cys-150), which enables GAPDH to interact with a nuclear localization signal (NLS)-containing protein Siah. This NLS is crucial for making the S-nitrosylated GAPDH-Siah complex to translocate to the nucleus [6]. Another study also showed a mechanism that facilitates nuclear translocation of the GAPDH-Siah complex via phosphorylation of Siah by apoptosis signal-regulating kinase 1 (ASK1) [16]. A follow-up study by Sen et al. [17] demonstrated that nuclear translocated GAPDH could interact with histone acetyltransferases P300 and CREB-binding protein (CBP). As a downstream of P300/CBP, although the mechanistic detail remains elusive, genes involving pro-apoptotic cascades are likely to be upregulated [17].

This nuclear GAPDH cascade has been reportedly activated in more than one genetic cell and animal models for neurological disorders [18,19]. A pharmacological intervention of this nuclear cascade inhibits the pathological outcome in a mouse model for merosin-deficient congenital muscular dystrophy type 1A (MDC1A) [19]. A more recent study reported that GAPDH *S*-nitrosylation at Cys-150 can also elicit acetylated tau (ac-tau) accumulation in response to cellular injury [20]. Following this study, Shin et al. [21] has demonstrated that traumatic brain injury (TBI) can induce GAPDH *S*-nitrosylation at Cys-150 and nuclear translocation of GAPDH, which leads to a coordinate activation of p300/CBP acetyltransferase and inhibition of NAD^+^-dependent histone deacetylase Sirtuin 1 (Sirt1). This cellular cascade, in turn, increases the amounts of the neuronal ac-tau and resultant neurogenerative processes. Pharmacological intervention targeting the nuclear GAPDH cascade inhibits tau acetylation and mitigates downstream consequences of brain injury [21]. Increased ac-tau has also been observed in postmortem brains from patients with Alzheimer's disease (AD), particularly those with history of TBI [21].

**Fig. 1:**
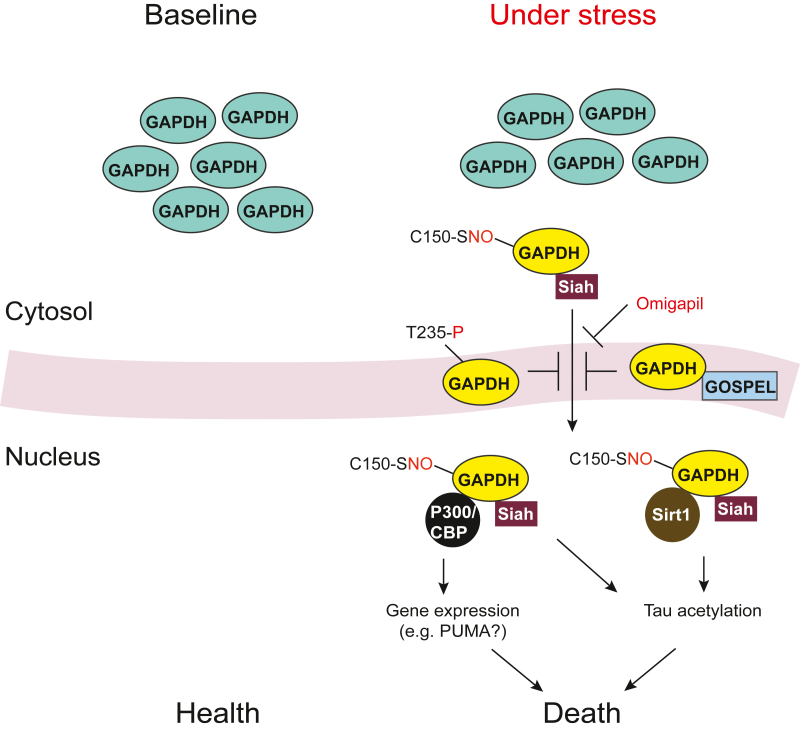
**Stress-induced GAPDH nuclear translocation toward cell death.** Triggered by *S*-nitrosylation of GAPDH on cysteine-150 in rat/−152 in human (C150–SNO-GAPDH), the GAPDH-Siah complex translocates to the nucleus, thereby interacting with transcriptional co-activators (P300/CBP) and co-repressor (Sirt1) and driving downstream effects toward cell death. Omigapil, the GAPDH-GOSPEL protein interaction, and phosphorylation on threonine-235 in rat/−237 in human (T235-P-GAPDH) can counteract this process.

In summary, GAPDH functions as an intracellular signaling molecule, with its nuclear translocation contributing to stress-associated pro-death mechanisms. Posttranslational modification at Cys-150 plays a crucial role in the initiation of this signaling cascade [[22], [23], [24]]. Interactions of GAPDH with transcriptional co-activators and co-repressor seems to be key nuclear events driving downstream effects (Fig. 1) [2].

### Nuclear GAPDH for cell survival in non-neuronal cells

Upon glucose starvation, Sirt1 is known to be activated, which leads to autophagy [25]. Genes transactivated by the Sirt1-associated cascade include autophagy related 12 (Atg12) [26]. Chang et al. [27] reported that AMP-activated protein kinase (AMPK), a sensor of cellular energy level, phosphorylates GAPDH at serine-120 in rat/serine-122 in human (Ser-120), which in turn triggers nuclear translocation of GAPDH and activates Sirt1. This mechanism may likely account for an initial finding of autophagic activation and cell survival by GAPDH, in which transactivation of Atg12 plays a crucial role [[27], [28], [29]].

**Fig. 2:**
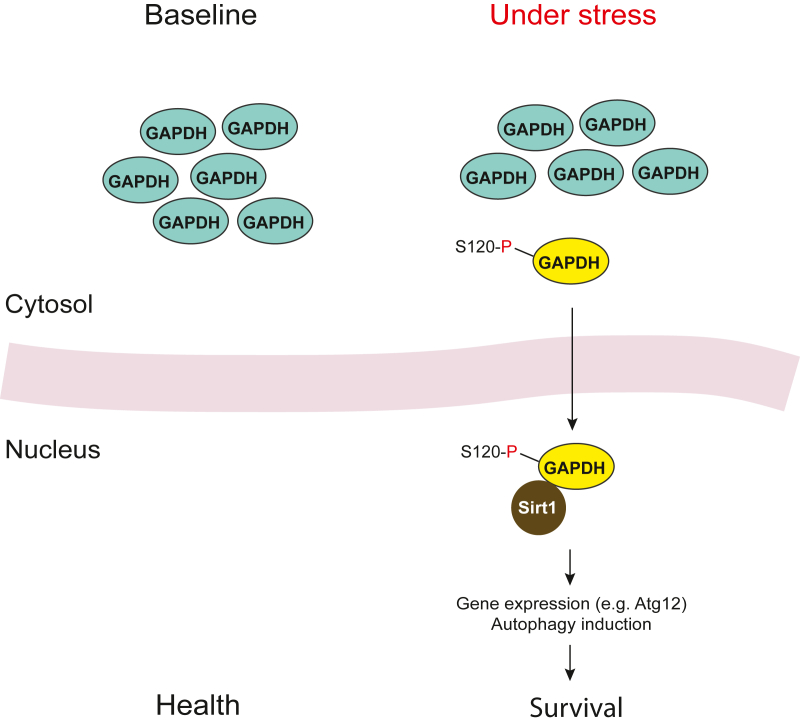
**Stress-induced GAPDH nuclear translocation toward cell survival.** Triggered by phosphorylation of GAPDH on serine-120 in rat/−122 in human (S120–P-GAPDH), GAPDH translocates to the nucleus, thereby interacting with transcriptional co-repressor Sirt1 and driving downstream effects toward survival, which may be associated with autophagy induction.

To the best of our knowledge, this mechanism has been tested and validated in non-neuronal cells. It remains to be elucidated whether and how the phosphorylation of GAPDH Ser-120 by AMPK and the subsequent interaction of GAPDH-Sirt1 may play a role for cell survival in neurons. In summary, in addition to the contribution to pro-death mechanisms, stress-induced GAPDH nuclear translocation (the nuclear GAPDH cascade) plays a role for a stress-associated pro-survival mechanism in different cellular contexts. Posttranslational modification at Ser-120 may play a role in the initiation of the signaling, and the interaction with a transcriptional co-repressor seems to be a key nuclear event (Fig. 2) [27].

### Nuclear GAPDH for cell dysfunction or cell fate changes in microglia, cardiac myocytes, and Arabidopsis

In addition to two lines of major studies on cellular mechanisms of nuclear GAPDH in response to stressors, more recent studies shed light on a broader l picture of stress-induced nuclear translocation of GAPDH and its biological outcomes.

In a mouse model under subacute cellular stress in which deficits in rule-shifting tasks testing cognitive flexibility were elicited, nuclear translocation of GAPDH was observed in microglia, but not in neurons, in the prelimbic cortex [30]. GAPDH nuclear translocation in this context is dependent on GAPDH *S*-nitrosylation at Cys-150 and the formation of GAPDH-Siah complex formation. Instead of microglial cell death, nuclear translocated GAPDH accompanies the induction of critical molecules involved in microglial influence on neuronal activities. Nuclear translocation of GAPDH selectively occurred in microglia is causal for adjacent neuronal activity changes and behavioral alternation. The pivotal role of the nuclear GAPDH cascade in stress-induced cognitive inflexibility has been proven by both cell type-specific molecular intervention and pharmacological intervention [30].

Stress-elicited GAPDH *S*-nitrosylation at Cys-150 followed by GAPDH-Siah complex formation and its nuclear translocation may also play a role in another pathological condition in the heart. In a mouse model for pressure-overload cardiac hypertrophy induced by transverse aortic constriction (TAC) *in vivo*, nuclear translocation of GAPDH was observed in hypertrophied cardiac myocytes [31]. Similar observations were seen in cultured cardiac myocytes using hypertrophic stimuli such as endothelin-1. In these conditions, no death of cardiac myocytes was observed. However, both cell type-specific molecular intervention and pharmacological intervention have shown that nuclear translocated GAPDH is causal for cardiac remodeling and resultant hypertrophy [31].

**Fig. 3:**
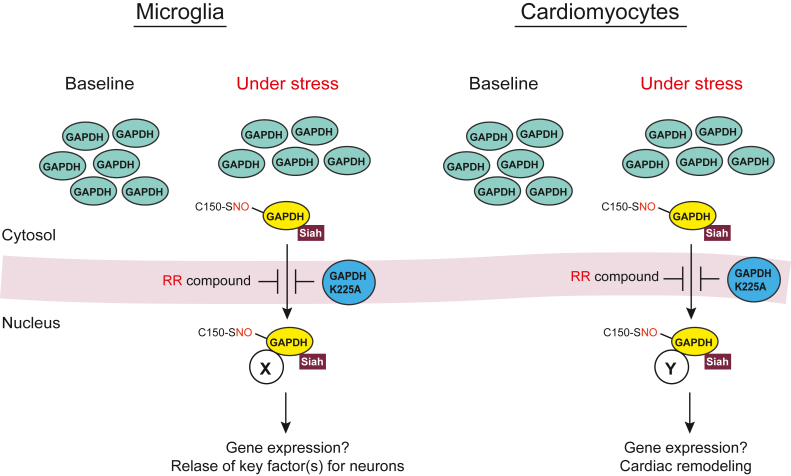
**Stress-induced GAPDH nuclear translocation in microglia and cardiomyocytes.** Triggered by *S*-nitrosylation of C150 GAPDH, the GAPDH-Siah complex translocates to the nucleus, thereby interacting with nuclear proteins (not fully identified and indicated as X and Y in this figure) and driving downstream effects. The RR compound can counteract this translocation. The replacement of lysine-225 with alanine in rat (−227 in human) (GAPDH K225A) disables the nuclear translocation of GAPDH.

The two studies in microglia and cardiac myocytes are intriguing, because stress-induced cell response mediated by GAPDH *S*-nitrosylation at Cys-150 does not result in cell death, instead leading to cell dysfunction of cell fate changes (Fig. 3). Altogether, the overall downstream outcome of GAPDH *S*-nitrosylation at Cys-150 seems to be diverse in a context-dependent manner. Furthermore, stress-induced GAPDH translocation to the nucleus is also known in Arabidopsis, in which nuclear GAPDH interacts with a transcription factor to promote the expression of heat-inducible genes and enhance heat tolerance [32].

### Counteracting mechanisms against nuclear translocation of GAPDH

Stress-induced nuclear translocation of GAPDH robustly impacts cell fate. Accordingly, several mechanisms that counteract its nuclear translocation have also been reported. To our best knowledge, a protein interactor of GAPDH, designated GOSPEL (GAPDH's competitor Of Siah Protein Enhances Life), may be the first report in this line [33]. GOSPEL binds to GAPDH, in competition with Siah, retaining GAPDH in the cytosol and preventing its nuclear translocation. GOSPEL is neuroprotective, as its overexpression prevents NMDA-glutamate excitotoxicity while its depletion enhances death in primary neuron cultures. This neuroprotective action against excitotoxicity has also been shown *in vivo*. Thus, GOSPEL is expected to physiologically regulate the viability of neurons and other cells by counteracting against GAPDH *S*-nitrosylation-mediated cell death [33]. In addition, Akt2 kinase suppresses GAPDH-mediated apoptosis in ovarian cancer cells via phosphorylating GAPDH at threonine-235 in rat/threonine-237 in human (Thr-235), thereby decreasing its nuclear translocation [34]. Furthermore, calcium and integrin-binding protein 1 (CIB1), previously known for its role in cell survival and proliferation, may also be implicated in a mechanism of counteracting nuclear GAPDH-dependent cell death [35]. Altogether, more than one mechanism may exist in counteracting nuclear GAPDH-mediated cell death (Fig. 1).

## What is known, and what is unknown for nuclear GAPDH?

Stress-induced nuclear GAPDH cascade has been extensively studied in the past a couple of decades as a major “moonlighting” feature of GAPDH. Indeed, as described below in greater detail, several efforts have taken place to lead the basic finding of nuclear GAPDH-mediated cell death to drug discovery and development. Nevertheless, the outcome has not been fully successful thus far. This may be partly because investigators pushed forward the translational process before realizing the complexity of the stress-induced nuclear GAPDH cascade.

Therefore, at present, it would be useful to organize scientific questions on nuclear GAPDH at the mechanistic levels (e.g., what is known and what is unknown about nuclear GAPDH) toward more successful drug discovery and development.

As far as we are aware, the following questions remain to be addressed (Table 1).

1) The mechanisms for nuclear translocation: obviously, there is more than one mechanism for this, such as posttranslational modification at Cys-150 and Ser-120 [6,17,27]. Note that Cys-150 modification-associated nuclear translocation has been frequently reported in the context of cell death and dysfunction, whereas Ser-120 modification-associated nuclear translocation in the context of cell survival. The modification at Thr-235 may be associated with the counteracting mechanism. Protein interactions with GOSPEL and ASK1 may also play a role in this translocation [16,33]. Nevertheless, their relationships and the coordinated regulation remain to be elucidated.
2) The function of nuclear-translocated GAPDH: after nuclear translocation, GAPDH has been reported to interact with several histone acetyltransferases and deacetylases, such as P300, CBP, and Sirt1 [17,21,27]. Their impact on gene transcription has been suggested. Nevertheless, the overall genome-wide landscape of gene transcription and epigenetics that are possibly affected by nuclear GAPDH remains to be elucidated. In addition to gene transcriptional regulation, what else does nuclear GAPDH play a role? Protein acetylation has been reported as described above [21]. How about glycolysis? Several reports have indicated that nuclear translocated GAPDH with Cys-150 nitrosylation loses its glycolytic activity [6]. It is also unclear whether stress-induced nuclear GAPDH is implicated in the role of GAPDH in telomeres. Many questions are still unanswered.
3) The cellular outcome elicited by stress-induced nuclear GAPDH: as described above, the outcome is diverse, which is not limited to cell death, but also cell survival and cell fate changes [6,21,27,30,31]. These differences may be partly accounted for by cell types. Nevertheless, a more comprehensive perspective is awaited to account for this diversity.
4) The types of stressors: such a high diversity in the cellular outcome may also be associated with different types of stressors. In the past multiple types of stressors have been tested across different cell types, complicating our understanding of context-dependent features of nuclear GAPDH. Systematic studies that tease out stress-type specificity from cell-type specificity are warranted.
5) The contrast of cell autonomous to non-cell autonomous implications: cell death is regarded as an unfavorable outcome in many contexts. Neuronal cell death is devastating. However, in the non-cell autonomous context, cell death in cancer cells leads to a favorable outcome. GAPDH has evolutionally acquired novel functions beyond its original role as a glycolytic enzyme, such as stress-induced nuclear translocation and resultant stress responses for the favorable outcome of the overall organisms. We may need to revisit the interpretation of the past data by paying attention to the contrast of cell autonomous to non-cell autonomous implications.

**Table 1:** Nuclear GAPDH: current understanding and unanswered questions.

Topic	Current understanding	Unanswered question
Mechanisms for nuclear translocation	Posttranslational modifications, Protein interactions (Siah, ASK1, and GOSPEL)	Coordinated regulation among triggers and counteracting factors for nuclear translocation
Function of nuclear-translocated GAPDH	Interaction with several histone acetyltransferases and deacetylases (P300, CBP, and Sirt1) and downstream cascades (gene expression change, protein acetylation)	Genome-wide landscape of gene expression and epigenetic changes Telomere-related function, Glycolytic function in the nucleus
Cellular outcome	Cell death, dysfunction, survival, fate changes	Mechanisms and contexts underlying the divergent outcomes
Types of stressors	Oxidative stress, inflammation, serum starvation, metabolic stress, genotoxin	Link between stressors, nuclear translocation and nuclear function of GAPDH, and cellular outcomes

## The potential of targeting nuclear GAPDH for neurotherapeutics

Although there is abundant room for further basic research regarding the “moonlighting” roles of GAPDH, including stress-induced nuclear GAPDH, several major efforts have been taken place for drug discovery and development targeting to neurological disorders, based on the basic discovery of nuclear GAPDH [[1], [2], [3],^8^,^9^,^36^].

### Promising compounds that block nuclear translocation of GAPDH in cell and animal models

Omigapil (dibenzo[b,f]oxepin-10-ylmethyl-methyl-prop-2-ynyl-amine), also called TCH346 or CGP3466B was originally developed as a structurally similar molecule to L-deprenyl or Selegiline, a monoamine oxidase (MAO) type B inhibitor [37]. L-deprenyl has been known to have a neuroprotective action [37]. Accordingly, Omigapil is neuroprotective without inhibiting MAO [38,39]. Mechanistically, Kragten et al. [40] showed the specific binding of Omigapil to GAPDH by affinity binding, affinity labeling, and the Biacore technology. Subsequently, Hara et al. [41] demonstrated that Omigapil prevents the nuclear translocation of GAPDH and the resultant cytotoxicity. Importantly, Omigapil can block the nuclear translocation and rescue cell death induced by trophic withdrawal in PC12 ​cells at 0.1 to 1 pico-molar order [41].

Beneficial effects of Omigapil have also seen in animal models for multiple diseases [38,39]. These include animal models for Parkinson's disease (PD) [[42], [43], [44]], amyotrophic lateral sclerosis (ALS) [45], and TBI [21], as well as experimental autoimmune encephalomyelitis (EAE) mouse model [46]. There reports have provided evidence that Omigapil is beneficial in a wide range of animal models for neurodegenerative conditions. Note that there was a negative report on Omigapil for ALS mouse models [47]. Furthermore, Omigapil was reportedly more effective in improving muscle function and strength when coupled with overexpression of the extracellular matrix molecule mini-agrin in MDC1A model mice [19,48]. The dual treatment indeed enhanced mechanical-load bearing ability and improved regeneration of muscle in the mouse model.

More recently, a different set of deprenyl structural analogues has been synthesized, among which (1R, 3R)-1,3-dimethyl-2-propargyl-1,2,3,4-tetrahydroisoquinoline (designated as RR compound) shows the highest potency for blocking the GAPDH-Siah protein interaction [30,31,49]. This RR compound has provided major beneficial effects in a stress-induced mouse model of cognitive inflexibility in which microglial nuclear GAPDH plays a pivotal role [30]. Furthermore, the RR compound also shows a major treatment effect against pressure-overload cardiac hypertrophy induced by TAC [31].

### Clinical trials with Omigapil

Two major studies took place with Omigapil for PD and ALS, respectively. However, both resulted in unsuccessful [8,9,36].

The phase II PD trial was in a double-blind, randomized, controlled design (Table 2) [8]. Patients presenting at 45 international movement disorder clinics with early untreated PD were assessed as part of this parallel-group, double-blind, randomized controlled trial. These 301 eligible patients were randomly assigned 12–18 months' treatment with Omigapil at a daily dose of 0.5 ​mg (n ​= ​78), 2.5 ​mg (n ​= ​79), or 10 ​mg (n ​= ​73), or placebo (n ​= ​71), followed by a 4-week washout period. The primary outcome measure was the time to the development of a disability requiring dopaminergic treatment. However, there were no significant differences between groups. Secondary outcome measures were the annual rate of change in the unified Parkinson's disease rating scale (UPDRS) and the Parkinson Disease Questionnaire (PDQ-39), a measure of quality of life. There were no differences between groups in the annual change in the UPDRS or PDQ-39 either. Altogether, the trial was unsuccessful [8].

**Table 2:** Clinical trials with Omigapil for PD and ALS.

Study	Study design	Participants	Treatment and duration	Primary outcome	Secondary outcome
Olanow et al. 2006 [8]	Parallel-group Double-blind Randomized	301 early stage idiopathic untreated PD patients Ages 31–86 years 45 locations in Europe, North and South America	Placebo or 0.5/2.5/10 ​mg Omigapil Oral administration, once daily 12–18 months treatment Followed by a 4 weeks washout period	Time to development of a disability requiring dopaminergic treatment. No significant differences between any of the Omigapil groups and the placebo group.	Annual rate of change in UPDRS (Part 2 and 3) and PDQ-39 scores Percentage of patients requiring symptomatic treatment within 12 months of starting Omigapil^a^ Omigapil-induced symptomatic effects^b^ Frequency and severity of individual adverse experiences and laboratory abnormalities^c^ No significant differences between any of the Omigapil groups and the placebo group
Miller et al. 2007 [9]	Parallel-group Double-blind Randomized	591 ALS patients Ages 21–80 years 42 locations in Europe and North America	Placebo or 1.0/2.5/7.5/15 ​mg Omigapil Oral administration, once daily 24–44 weeks treatment	Rate of change in the revised ALSFRS-R No significant differences between any of the Omigapil groups and the placebo group	Survival, pulmonary function, and MMT No significant differences between any of the Omigapil groups and the placebo group

PD, Parkinson's disease; ALS,amyotrophic lateral sclerosis; UPDRS, unified Parkinson's disease rating scale; PDQ, Parkinson Disease Questionnaire; ALSFRS-R, ALS functional rating scale; MMT, manual muscle testing.

a Drug was needed by 23 (32 ​%) patients in placebo group, 26 (34 ​%) in 0.5 ​mg group (difference compared with placebo 2 ​%, 95 ​% CI -13.8 to 16.5), 30 (38 ​%) in 2.5 ​mg group (difference 6 ​%, −9.7 to 20.8), and 24 (33 ​%) in 10 ​mg group (difference 1 ​%, −14.8 to 15.8).

b Omigapil-induced symptomatic effects were determined by washin (change in UPDRS score between baseline and 4 weeks of treatment) and washout (change in UPDRS score between final treatment visit and 4 weeks washout visit) assessments.

c The overall frequency of adverse events was higher in the placebo group (85 ​%) than in the 0.5 ​mg group (81 ​%; difference −4 ​%, 95 ​% CI -15.9 to 8.4), in 2.5 ​mg group (76 ​%; difference −9 ​%, −21.2 to 4.1), and in 10 ​mg group (70 ​%; difference −15 ​%, −28.1 to −1.2). Serious adverse events occurred in 4 ​% of patients treated with Omigapil compared with 7 ​% in the placebo group (difference −3 ​%, −9.2 to 3.8). None of the adverse events or serious adverse events were judged to be related to the study drug.

Phase II/III randomized trial of Omigapil also took place in patients with ALS (Table 2) [9]. Five hundred ninety-one ALS patients were enrolled at 42 sites in Europe and North America, and they were randomly assigned in a double-blind fashion to receive either placebo or one of four doses of Omigapil (1.0, 2.5, 7.5, or 15 ​mg/day) administered orally once daily for at least 24 weeks. The primary outcome measure was the rate of change in the revised ALS functional rating scale (ALSFRS-R). Secondary outcome measures included survival, pulmonary function, and manual muscle testing (MMT). There were no differences in baseline variables. There were no significant differences between placebo and active treatment groups in the mean rate of decline of the ALSFRS-R or in the secondary outcome measures (survival, pulmonary function, and MMT). The trial revealed no evidence of a beneficial effect of Omigapil on disease progression in patients with ALS [9].

More recently, based on animal model data [19,48], the trials for congenital muscular dystrophy were started. The phase I trials with Omigapil were conducted to test pharmacokinetics and tolerability at a range of doses in patients to achieve a desired predetermined target area under the plasma concentration-vs-time curve in patients and to explore the feasibility of conducting disease-relevant clinical assessments [50].

### Lessons from past clinical trials

Although deprenyl-derivatives such as Omigapil and RR compound have provided a high level of the preclinical promise in cell and animal models, there is a major gap between this and the clinical outcome with Omigapil. There may be multiple reasons. For example, these animal models represent disease-associated phenotypes in a relatively acute manner, whereas PD and ALS are chronically progressed. Patients even under the same diagnosis are heterogeneous, and the relevant patient subgroup matching with the experimentally validated condition might not be selected. The observation period might be too brief to see a therapeutic effect. Among them, the lack of biomarkers directly driven from the GAPDH-associated mechanism may also be a reason for the failure. Nevertheless, Omigapil is relatively tolerable in humans and highly selective at the pharmacological levels. Thus, a better mechanistic understanding of stress-induced nuclear GAPDH cascade, development of mechanism-driven biomarkers, and relevant patient selection based on the markers may provide hope of repurposing Omigapil and developing related compounds.

## Summary

In this review article, we highlighted the roles of stress-induced nuclear GAPDH among multiple roles of this moonlighting protein (Table 3). Previously, because of the existence of a highly selective compound targeting the nuclear translocation of GAPDH with the preclinical premise, clinical trials for PD and ALS took place and turned out to be unsuccessful. Here we have collected several scientific updates of stress-induced nuclear GAPDH even after the clinical trials, deciphering more complexity of the mechanism. Based on the frontline knowledge, we made a discussion on this cascade and its potential for future translation.

**Table 3:** Moonlighting protein GAPDH.

Glycolytic pathway
Healthy conditions: global
Healthy conditions: locally associated with cytoskeleton
Healthy conditions: membrane-associated proteins
Cancers (in association with Warburg effect)
Non-glycolytic pathway
**Stress-induced nuclear translocation, affecting nuclear signaling for cell death, survival, and cell fate changes**
Interaction with RNAs (including IFN-γ mRNA interaction for T cell function control)
Possible regulation of telomeres
Interaction with mitochondrial proteins for functional alteration

Bold: the topic of this review article (also see Fig. 1, Fig. 2, Fig. 3).

## Disclosures

AS issued a patent (US20150218103A1) entitled “Gapdh cascade inhibitor compounds and methods of use and treatment of stress induced disorders including mental illness.” Except this, other authors have nothing to declare.

## Author contributions

Parimala Vedula, conduct extensive literature search, participate in the discussion of the manuscript preparation, and participate in writing the manuscript and preparing the figures and table.

Koko Ishizuka, participate in writing the manuscript and preparing the figures and table, as well as conduct extensive literature management.

Arisa Hayashida: prepare a figure, conduct extensive literature search, and participate in the discussion of the manuscript preparation.

Kota Sueo, conduct extensive literature search, and participate in the discussion of the manuscript preparation.

Akira Sawa: write the manuscript, prepare the figures and table, as well as lead the discussion of the manuscript preparation.

## Declaration of competing interest

The authors declare that they have no known competing financial interests or personal relationships that could have appeared to influence the work reported in this paper.
